# Analysis of Polyadenylation Signal Usage with Full-Length Transcriptome in *Spodoptera frugiperda* (Lepidoptera: Noctuidae)

**DOI:** 10.3390/insects13090803

**Published:** 2022-09-02

**Authors:** Liying Fang, Lina Guo, Min Zhang, Xianchun Li, Zhongyuan Deng

**Affiliations:** 1 School of Agricultural Sciences, Zhengzhou University, Zhengzhou 450001, China; 2 School of Life Sciences, Zhengzhou University, Zhengzhou 450001, China; 3 Department of Entomology, BIO5 Institute, University of Arizona, Tucson, AZ 85721, USA

**Keywords:** *Spodoptera frugiperda*, polyadenylation signal, alternative polyadenylation, poly(A)

## Abstract

**Simple Summary:**

RNA polyadenylation is an important process in mRNA maturation. The process is controlled by various cis-acting elements surrounding the cleavage site, and their binding factors. Recently high sequencing technology especially full-length transcriptome provides a large amount of sequencing data for us to explore the variations of poly(A) signals, alternative polyadenylation (APA) in *Spodoptera frugiperda.* We studied 50,616 polyadenylation signals using full-length transcriptome and EST data. These data show that 51.64% of the 50,616 pA sites had the conserved AAUAAA hexamers, while 10.13% of pA sites had none of the AAUAAA-like hexamers. Among these genes, more than 64.76% have more than one pA site. Our results also support that APA plays a significant role in increasing transcriptome diversity and gene expression regulation in *Spodoptera frugiperda*. Our dataset was the first polyadenylation signal analysis in the Lepidoptera and would provide a theoretical basis for pest control.

**Abstract:**

During the messenger RNA (mRNA) maturation process, RNA polyadenylation is a key step, and is coupled to the termination of transcription. Various cis-acting elements near the cleavage site and their binding factors would affect the process of polyadenylation, and AAUAAA, a highly conserved hexamer, was the most important polyadenylation signal (PAS). PAS usage is one of the critical modification determinants targeted at mRNA post-transcription. The full-length transcriptome has recently generated a massive amount of sequencing data, revealing poly(A) variation and alternative polyadenylation (APA) in *Spodoptera frugiperda*. We identified 50,616 polyadenylation signals in *Spodoptera frugiperda* via analysis of full-length transcriptome combined with expression Sequence Tags Technology (EST). The polyadenylation signal usage in *Spodoptera frugiperda* is conserved, and it is similar to that of flies and other animals. AAUAAA and AUUAAA are the most highly conserved polyadenylation signals of all polyadenylation signals we identified. Additionally, we found the U/GU-rich downstream sequence element (DSE) in the cleavage site. These results demonstrate that APA in *Spodoptera frugiperda* plays a significant role in root growth and development. This is the first polyadenylation signal usage analysis in agricultural pests, which can deepen our understanding of *Spodoptera frugiperda* and provide a theoretical basis for pest control.

## 1. Introduction

RNA polyadenylation is crucial to gene expression in eukaryotes. This mechanism is achieved by adding a 3′poly(A) tail to the 3′ end of mRNA precursor cleavage site (CS). It not only affects the stability of mRNA, but also has great significance in post-transcriptional processes such as mRNA translation and trans-nuclear membrane transport [1,2,3,4,5]. It can be affected by a variety of factors, including self-carrying elements and abundant trans-acting protein factors. Most importantly, two core cis-elements are the sites for protein recognition. The first core cis-element is known as the polyadenylation site (PAS), which is a highly conserved AAUAAA hexamer motif and located 10–35 bp upstream of the 5′end of the cleavage site, and the other is a variable sequence rich in U/UG located in 15–30 bp downstream of the 3′ end of the cleavage site [6,7,8,9]. During polyadenylation, they are bound by two protein complexes. Cleavage and polyadenylation specificity factors (CPSF) bind to protein complex via PAS, and U/UG-rich element is bound by cleavage stimulation factors (CstF) [10]. Algorithmic analysis of transcripts with poly(A) tails reveals that the hexamer motifs of PAS in humans, mice, *Schmidtea mediterranea*, and *Drosophila melanogaster* are not constant; 40–59% of PAS in mRNAs use the AAUAAA as motifs, 25–40% use mononucleotide variants of AAUAAA as motifs, 13–25% have no recognizable canonical motif [10,11,12,13].

Many genes have multiple polyadenylation (PA) sites, which adds uncertainty to mRNA cleavage. After variable polyadenylation (APA) of pre-mRNA, different types and lengths of mRNA subtypes will be formed, including mRNA with 3′ noncoding regions (3′UTRs) of different lengths and transcripts with different coding sequences [6,14]. No matter which mRNA isoform is formed, it will have a great impact on the mRNA itself and protein translation. The difference in APA coding sequence will lead to the change of the C-terminal amino acid sequence of a protein, causing proteins to have different functions [11,15,16,17]. Even if the amino acid sequence remains, differences in the sequence of UTR-APA will affect the stability of mRNA and change the binding ability of RNA-binding protein or microRNA [9,18]. Therefore, the cis-element of a specific gene with multiple PA sites is of great significance for the transcription of mRNA precursors, and different PA sites determine how the gene is regulated [19,20].

So far, the first insect that has been used by scientists for PAS analysis is *Drosophila melanogaster* [12,21,22]. *Drosophila melanogaster* polyadenylation signal has a diffuse A-rich region, including the AAUAAA motif, extending to 40 nt upstream from the cleavage site. In both mammals and the fly, there is a U-rich region just downstream of the cleavage site, corresponding to the CstF binding region. In addition, the distribution seems to be similar, with means of 17, 16, and 17 nt in human, mouse, and fly respectively. Furthermore, insects are the largest group of animals, and their populations have diversity [23]. PAS usage may vary in insects, so more insect species need to be identified and analyzed for PAS.

*Spodoptera frugiperda* belongs to the Lepidoptera (Noctuidae). It is an omnivorous and gluttonous agricultural pest which can damage more than 353 kinds of grain and cash crops in nearly 76 families [24]. Meanwhile, *Spodoptera frugiperda* originated from America, and spread to Africa in 2016 and China in 2019, causing great damage, especially to corn and rice [25,26]. With the rapid development of high-throughput sequencing technology in the past 10 years, its genome has been sequenced and more than 9 genome versions have been published [27,28,29,30,31,32,33,34]. However, the genome annotated work is not ideal. Difficulties arise due to insufficient genomic data on the 3′-end processing of annotated protein-coding genes [32].

Here, we utilize the systematic mapping of the raw data from *Spodoptera frugiperda* ESTs and full-length transcriptome data to the genomes and then analyze PAS and DSE signals with a general process. Our research analyzes the main traits of mRNA PAS signal and APA genes. Therefore, our poly(A) study in *Spodoptera frugiperda* will deepen the understanding of RNA post-transcriptional regulation and assist in the annotation of the *Spodoptera frugiperda* genome.

## 2. Materials and Methods

### 2.1. The Source of the Sequencing Data

Full-length transcriptome data of *Spodoptera frugiperda* were downloaded from the NCBI Sequence Read Archive database (https://www.ncbi.nlm.nih.gov/sra/, accessed date: 19 March 2022), and the accession numbers are SRR14569375.1, SRR14569378.1, and SRR14621792.1 [35]. The full-length transcriptome data from SRA database was used PacBio single-molecule real-time (SMRT) sequencing aimed at revealing the full-length transcriptome profiling of the FAW larval brain to obtain detoxification genes [35]. EST (Expressed Sequence Tag) data of *Spodoptera frugiperda* were searched and downloaded from NCBI (https://www.ncbi.nlm.nih.gov/nuccore/, accessed date: 1 August 2021), and sequence is presented in Dataset S1.

### 2.2. Identification of Poly(A) Sites

In this process, putative novel polyadenylation sites were identified. To achieve it, we first ascertained all sequencing reads starting or ending with adenine or thymine that had a minimum length of 10 nt, which followed Zhang [2] and Tian’s [11] process. Next, we removed the terminal As or Ts. We then extracted 200 bp sequences upstream of the polyadenylation site. After the above steps, we aligned both EST data and full-length transcriptome data with the *Spodoptera frugiperda* genome (ZJU_Sfru_1.0 (NCBI project 6098571, GCA_011064685.1)) through bowtie2 (http://bowtie-bio.sourceforge.net/bowtie2/index.shtml, accessed date: 1 June 2018, Version 2.4.3, Selectable parameter: -p 10-score-min L, −0.3, −0.3 -v 1). The reads were mapped uniquely to the genome in this phase and then utilized samtools (http://www.htslib.org/, Version 1.1.0, accessed date: 1 June 2018) to determine the specific base at which cleavage took place. To remove apparent cleavage sites potentially resulting from sequencing errors (As and Ts in the genome are erroneously considered as poly(A) tails), we deleted cleavage sites where the 10 bp downstream cleavage site genomic regions contained equal to or more than 9 As or Ts. Ultimately, we extracted the 100 bp upstream and downstream sequence for further research.

### 2.3. Polyadenylation Signal Search and Analysis

We analyzed single-nucleotide profiles using 100 bp upstream and 100 bp downstream of the cleavage site. Next, we did an analysis for all 6 hexamer motifs from 0–50 bp and the data of these motifs included there, along with the number of related genes. After completing this work, we got the top 18 motifs that had only one nucleotide difference. Then, the AATAAA and AATAAA with one bp variation frequency at the upstream of cleavage site were further examined. Additionally, the cleavage site downstream elements (such as U/GU rich element) were explored simultaneously at 100 bp downstream of the cleavage site. The perl script is presented in Script S1.

### 2.4. APA Analysis in the Spodoptera frugiperda Genome

To analyze the global distribution of APA in *Spodoptera frugiperda,* the gene annotation information of *Spodoptera frugiperda* was downloaded (http://v2.insect-genome.com/Organism/715, accessed date: 1 March 2022) [32]. We compared the CS position with the gene location on the chromosome, and the gene containing two or more cleavage sites means the gene was potentially regulated by alternative polyadenylation. After this, the gene containing the PAS number was analyzed.

### 2.5. PAS Information Annotation

The PAS positions in the chromosome were obtained by extracting the mapped data of the poly(A) tail reads. Next, the gene number in each of the 32 chromosomes was then extracted using the genome annotation file, and PAS in different gene positions were also examined.

### 2.6. Data Analysis

Chi-square (χ^2^) test was used to determine if the frequency of A, C, G, and U nucleotide around the polyadenylation cleavage site is significantly different from the expected A, C, G, and U frequency in *Spodoptera frugiperda* genome (GC content in *Spodoptera frugiperda* genome was 36.4%, G or C frequency should be 18.2%, A or U frequency should be 31.8%).

## 3. Results

### 3.1. Identification of Polyadenylation Sites

**Figure 1:**
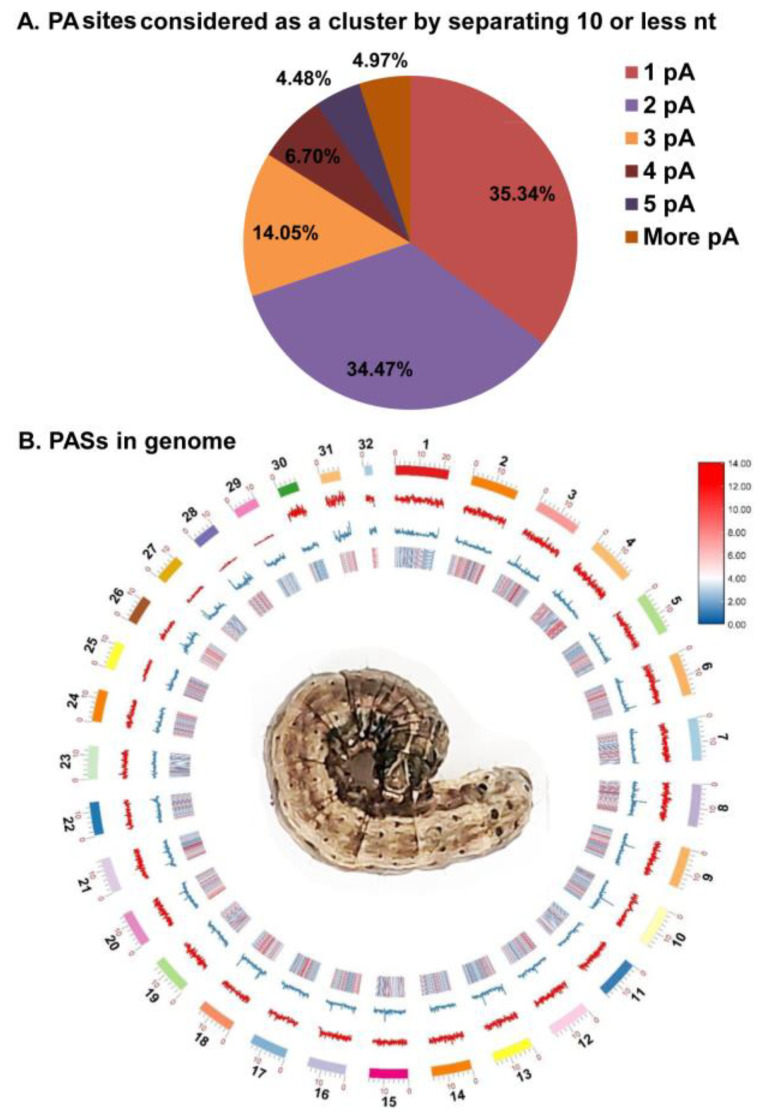
Identification of poly(A) sites (pA sites) in *Spodoptera frugiperda*. (**A**) A cluster of one or more pA sites with an inter-node separation of 20 or less nt. (**B**) The diagram shows pA site density across 32 chromosomes related to the gene, GC%, and repeat content. According to the sequence from outside to inside, the graph shows: ideogram of chromosomes in Mb, line graph of pA sites distribution, line graph of GC distribution as percentage to total bases number, gene density heat map. Red represents the highest value and blue represents the lowest value.

The raw ESTs and full-length transcriptome for *Spodoptera frugiperda* contained a total number of 65,423 ESTs and 7,510,007,630 raw full-length transcriptome reads (Table 1). Next, we removed the low-quality reads which started or ended with As or Ts less than 10, and finally we obtained a sum of 35,853 EST sequences and 375,500,381 full-length reads with poly(A) tails (started or ended with As or Ts more than 10). These reads with poly(A) tails were then mapped to the *Spodoptera frugiperda* genome (ZJU_Sfru_1.0 (NCBI project 6098571, GCA_011064685.1)), yielding a total of 10,757,513 uniquely mapped cleavage sites (28,931 from 35,853 EST reads and 10,728,582 from 375,500,381 full-length reads). A total of 50,616 pA sites (Table S1, 10,245 from 28,931 EST reads and 40,371 from 10,728,582 full-length reads) were discovered when internally primed cleavage sites were discarded (at least eight consecutive A in the genome aligned downstream of the cleavage site) and those sites that were closely separated by less than or equal to 20 bp were merged (Table 1, Figure 1B). There was only one cleavage site found in 35.34% of them and no heterogeneity was observed (Figure 1A). Moreover, two (34.47%) and several (30.19%) alternative cleavage sites were discovered at the remaining pA sites (Figure 1A).

### 3.2. Nucleotide Bias Close to the Cleavage Sites

**Figure 2:**
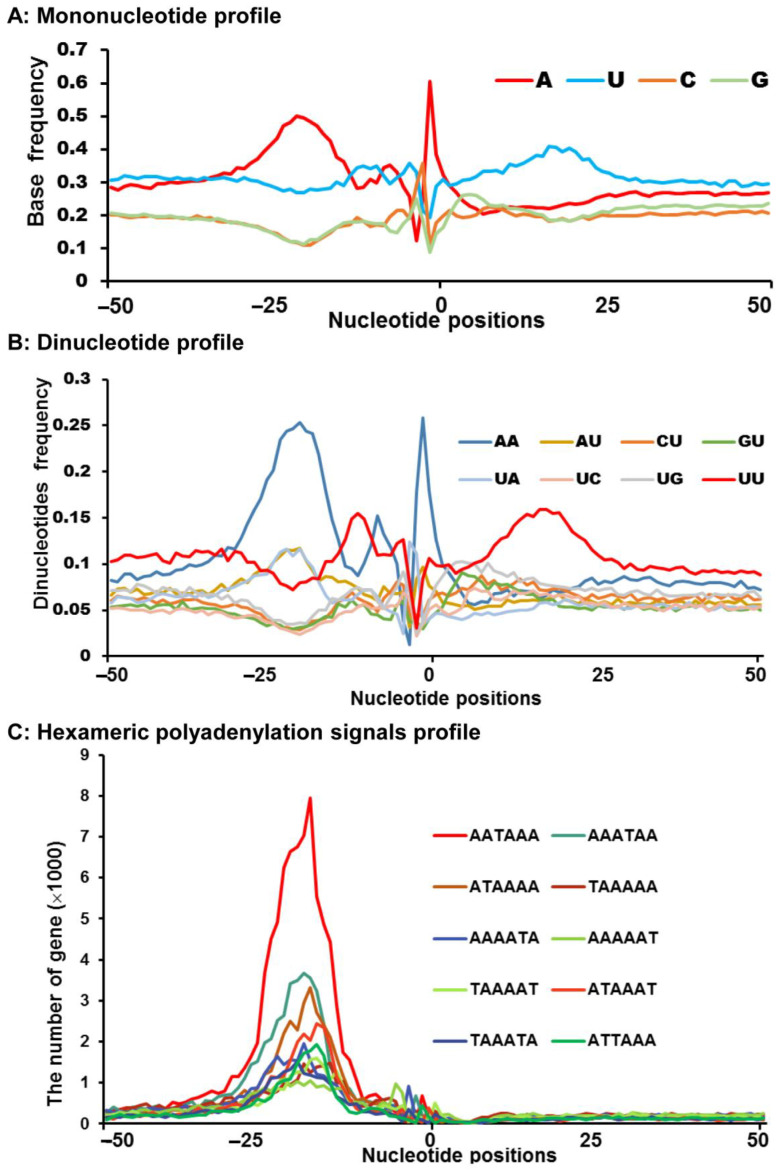
Sequence characterization around the cleavage site. (**A**) Distribution frequency of A, C, G, and U within 50 bp of the cleavage site of *Spodoptera frugiperda*. (**B**) Distribution frequency of the dinucleotides within 50 bp upstream and downstream of the cleavage site. Only top 8 dinucleotides were shown. (**C**) The number of hexamers within the 50 bp of the cleavage site. Only the top 10 motifs were shown. The number of genes is shown on the *Y*-axis. The position zero is the CS.

To more clearly show the above core cis-elements, including 50,616 polyadenylation sites, we analyzed the single nucleotide profiles of 50,616 polyadenylation sites within 100 bp upstream and downstream of the corresponding cleavage sites. Under completely random conditions, A, U, C, and G, the frequency of four nucleotides appearing in every position of this region should be 25%. Considering the GC content in *Spodoptera frugiperda* genome was 36.4% (G or C frequency should be 18.2%), and A and U frequency were close to 63.6% (A or U frequency should be 31.8%). However, it was discovered that the frequency of U remained close to 30%, except for position 0 (the cleavage site), where it was found to be about 22.37% (no significant, *p* = 0.15). There were three frequency peaks for U and they were respectively located at position 3–27, −6 to −4, and −9 to −12. Coincidentally, the downstream core U/UG-rich sequences that CstF can particularly identify during polyadenylation coincided with the rise to the 3–27 U peaks. Except for the peaks corresponding to 0, −32 to −14, and −7 to −5, and a trough corresponding to 3–27, the probability of A at other positions remained around 25% and most regions were lower compared to the U. The A-rich region at position −32 to −14 corresponding to the hexamer element like core AAUAAA bound by CPSF. Furthermore, the CFI and CFII complex identified the cleavage site indicated by the A peak at position 0 (frequency 62.37%, *p* = 0.0001544). In comparison, C and G remained below 25% and close to 20%, except for positions −1 (C and G) and 4–5 (G only), in which they reached 25% or slightly higher (Figure 2A). The downstream U/GU-rich element corresponded to the G and U peaks at positions 4–5 and 3–27, respectively.

Because in many eukaryotes CA and UU/GU were abundant at positions −1 and 0 and 15–30 bp 3′ of the cleavage site, respectively [8,10], we analyzed 16 types of dinucleotide profiles of the 50,616 pA sites within 100 bp upstream and downstream of their cleavage sites (Figure 2B and Figure S1A). Moreover, AA-enriched positions were comparable to single nucleotide A in tendency, according to the dinucleotide profiles (Figure 2B and Figure S1A). In addition, the AA rich region showed an excessive expression with a frequency of >0.08 from position 23–100, while other dinucleotide such as AC, AG, and AU had no significant tendency (Figure S1A). What is more, the region −1 to 1 occurred a sharp CA peak and proved that it was a cleavage site. However, the CC, CG, and CU had no such condition. Although the tendency of the GA was similar to that of the CA, it has a smaller proportion. Meanwhile, a sharp peak for UA appeared in the region of −1 to 1, and a slightly wider peak appeared in the region of −14 to −24. In accord with the downstream U/GU-rich element, the UU rich significantly occurred at regions from −4 to −13 and 10 to 26, with a focus on 14 to 22. Moreover, the frequency of the remaining dinucleotides at positions 1–100 was between 0.03 and 0.08, which appeared random (Figure 2B and Figure S1A).

### 3.3. Sequence Motifs near the Cleavage Sites

A total of 50,616 pA sites previously identified were screened for all potential 6-base hexamers 100 bp upstream or downstream of the cleavage sites in order to determine the different forms of polyadenylation signals present in *Spodoptera frugiperda* (Figure 2C and Figure S1B). A hexamer’s scattered distribution at low frequency would be viewed as noise that happens at random. Hexamer signals, on the other hand, were defined as high-frequency peaks in a hexamer distribution. According to the above criteria, compared to the other hexamers, the motif AAUAAA showed the highest sharp peak at position −11 to −30. This phenomenon suggested that AAUAAA is the most important polyadenylation signal motif. Moreover, while the sharp peaks at positions −11 to −30 were present in the single-nucleotide variations of the motif AAUAAA, such as AUUAAA, ACUAAA, and AGUAAA, they were blunter and their values were not as high as those of AAUAAA. Except for these, the other variants, including UAUAAA, AAUAUA, AAUACA, CAUAAA, GAUAAA, and AAUAGA, also displayed peak values at position −11 to −30, which indicated that they were PAS as well, but had lower kurtosis than the above-mentioned hexamers. In addition, there was also a peak upstream of the cleavage site for the AAAUAA and AUAAAA, though they might be affected by the AAUAAA motif due to a shared portion. The variation UUUUUU could not be considered a real PAS since, unlike other hexamers, it exhibited no distinct peak and merely a dispersed distribution.

### 3.4. Usage Frequencies of Core Hexamers Polyadenylation Signal

In the 40 nt upstream of the 50,616 collected poly(A) sites, 89.87% of them contained at least one type of classical PAS hexamer (Table 2). In addition, it showed that the usage frequency of the most fundamental motif AAUAAA was 51.64% in *Spodoptera frugiperda,* lower than that of humans and mice [11]. Except for the most common hexamer AAUAAA, we found that single-nucleotide variant AUUAAA was the second most commonly used hexamer (12.15%). The 14 PAS motifs in the table were unable to be found in the remaining 10.13% of the pA sites called none, showing a higher ratio than humans.

### 3.5. pA Sites Distribution in the Spodoptera frugiperda Genome

**Figure 3:**
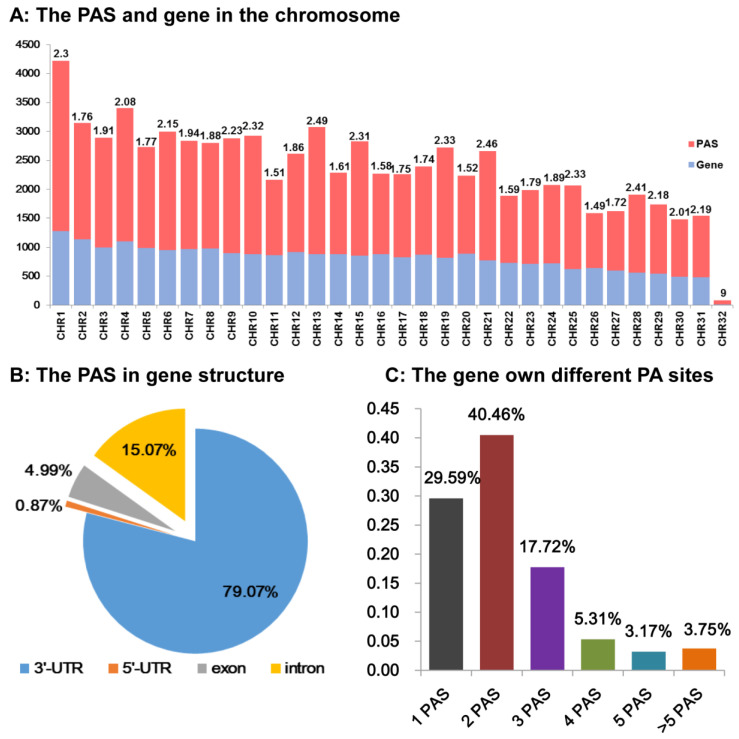
The APA in *Spodoptera frugiperda*. (**A**) The gene number, PAS site number, and ratio of PAS site to the gene on a particular chromosome. We analyzed the distribution of genes and PAS on the chromosome. We chose the color red to symbolize the PAS number and the color blue to represent the gene’s number on the chromosome. (**B**) The position of the PAS inside the gene structure. We investigated the frequency of PAS in the 3′UTR, 5′UTR, exon, and intron of genes. (**C**) The proportion of *Spodoptera frugiperda* genes that have multiple poly (A) sites. *Y*-axis was the ratio of gene with PA sites to total gene. We counted the number of PAS sequences present in each annotated gene and divided them into six categories: 1, 2, 3, 4, 5, and >5 PAS sequences.

Firstly, the distribution of the 50,616 pA sites above and the 25,699 annotated protein-coding genes [32] on the 32 autosomes and sex chromosomes were compared (Figure 3A). In the 32 chromosomes, the number of pA sites was much higher than that of genes. The pA sites to gene number ratio was from 1.49 to 2.49 in all chromosomes except for the Chr32 chromosome, and the Chr32 pA sites to gene number ratio was up to 9. In general, the number of pA sites was roughly correlated with that of genes (Figure 3A). Chr32 was the W chromosome, 8 genes or transcripts and 72 pA sites were observed, which was significantly lower than other chromosomes in genes and pA sites number. Then, we also found that 69.26% (35,055 out of 50,616) of the pA sites were distributed in 15,218 (out of the 25,699) annotated protein coding genes and 30.74% (15,561) were located in the regions of non-annotated genes, respectively (Figure 3B,C).

Among the 35,055 pA sites, which were associated with 15,218 genes, most of them (number and percentage of 79.07%) were found in 3′UTR s (Figure 3B). The second and third pA site-containing sections, with 15.07% and 4.99% pA sites, respectively, were home to the introns and exons of these genes. We also found 0.87% pA sites in the 5′UTR region of these genes (Figure 3B). Among the 25,699 annotated transcripts mapped with pA sites, excluding unknown genes, 70.41% of them have two or more alternative pA sites (Figure 3C), resulting in 3′ encoded or UTR sequences of variable length.

### 3.6. GO Analysis and KEGG Analysis of Alternative Polyadenylation Gene in Spodoptera frugiperda

**Figure 4:**
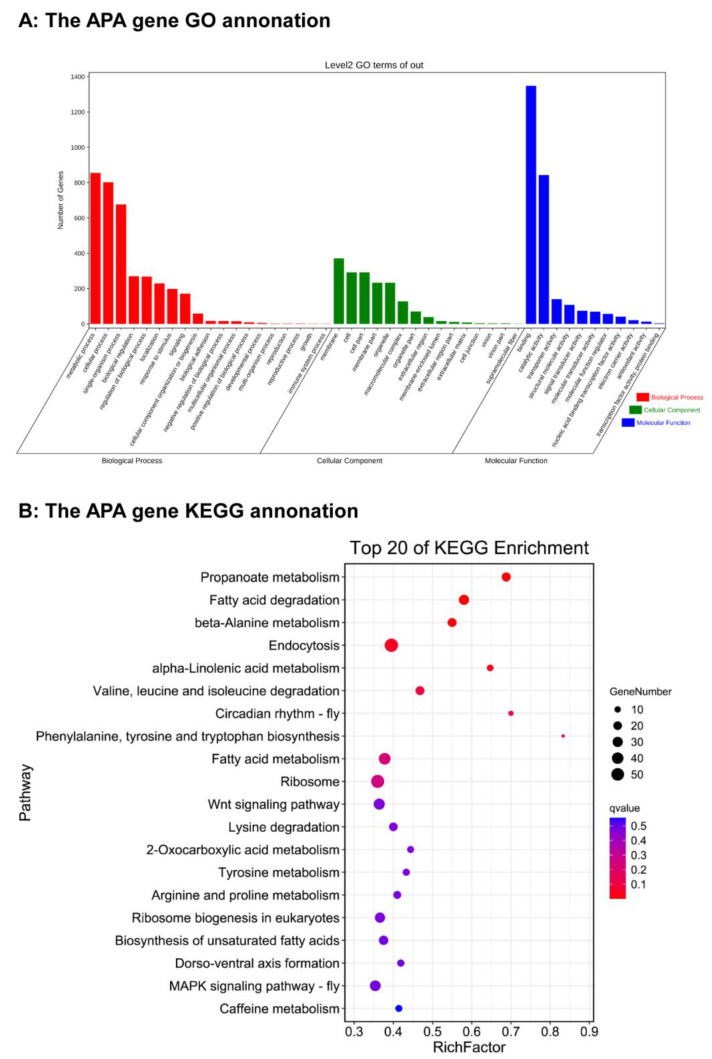
The alternative polyadenylation gene annotation. (**A**) The genes with 2 or more polyadenylation signals were applied for enrichment tests. (**B**) KEGG enrichment of genes with two or more PAS pathways is shown in a scatter diagram. The top 20 pathway names are represented on the *Y*-axis, and the *X*-axis shows the proportion of annotated genes to all annotated genes in a pathway. The colors of dots represent the different value. Red is the lowest value and blue is the highest value The size of each dot represents the gene number.

In order to better analyze the biological functions and distribution characteristics of the APA gene of *Spodoptera frugiperda*, we used bioinformatics methods to conduct GO analysis and KEGG analysis on the screened genes with two or more PA sites (accounting for 70.41%) (Figure 4, Figures S2 and S3). In the column of biological processes, the number of genes enriched by three biological processes, namely, metabolic process, cellular process, and single organization process, is the most prominent, with more than 600 (Figure 4A). In the cell localization column, APA genes are mainly concentrated in five parts: membrane, cell, cell part, membrane part, and macromolecular complex, of which the number of genes concentrated in the membrane is the largest (Figure 4A). In the column of molecular function, binding and catalytic activity enrich the most genes. It can be seen that the protein encoded by the APA gene often performs specific biological functions such as binding protein and catalytic protein (Figure 4A).

In KEGG analysis, we selected the top 20 genes with the highest enrichment factor as a reference. The results showed that the number of genes enriched in endocytosis, ribosome, ribosome biology in eukaryotes and other pathways was the largest, but the degree of enrichment was relatively low (Figure 4B). The phylalanine, tyrosine, and tryptophan biosynthesis pathways were the most enriched (Figure 4B). It can be seen from the results that most of the 20 KEGG pathways are metabolic pathways, indicating that the protein encoded by the APA gene is of great significance for cell metabolism, which is consistent with the results of the analysis.

Through GO analysis and KEGG analysis, it can be seen that the protein encoded by the APA gene bears important biological functions and strictly controls the process of cell metabolism. The in-depth study of APA mechanisms has a guiding significance for regulating metabolism and changing traits.

### 3.7. Different Alternative Polyadenylation Types

**Figure 5:**
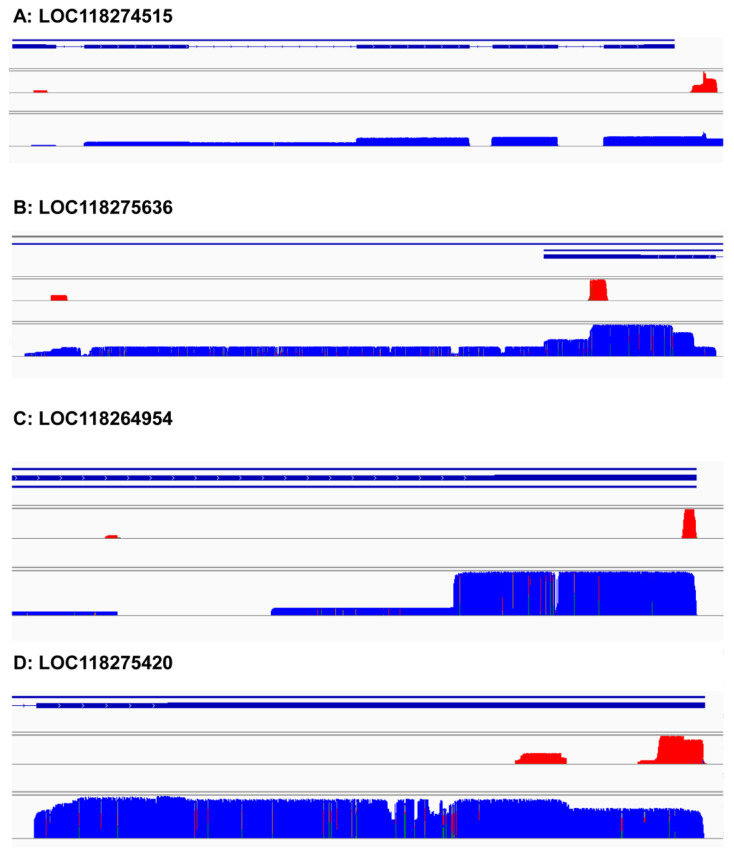
Different types of APA occur in *Spodoptera frugiperda*. (**A**) LOC118274515, a short-chain specific acyl-CoA dehydrogenase. The first pA site is in 5′UTR, another one is in 3′UTR. (**B**) LOC118275636, probable peroxisomal acyl-coenzyme A oxidase 1. The first pA site is in ORF, another one is in 3′UTR. (**C**) LOC118264954, cadherin-related tumor suppressor-like. The first pA site in an intron, another one in 3′UTR. (**D**) LOC118275420, Acyl-CoA dehydrogenase. Both pA sites are in 3′UTR. The red peak shows the position of poly (A) containing short reads, and the blue is the sequence of the full-length transcript.

Variable tailing can be divided into many types according to different positions of genes. Some will change the protein sequence, and some will not change the protein sequence but will change the RNA regulatory elements. We selected four genes (gene number: LOC118274515, LOC118275636, LOC118264954, LOC118275420) to explore the regulation of variable tailing on genes. LOC118274515, a short chain specific acyl CoA dehydrogenase, has a variable tailing site in the 5′UTR, primarily one in the 3′UTR. If the variable tailing of 5′UTR is mainly used, this gene will only transcribe some non-coding transcripts (Figure 5A). LOC118275636 was most likely peroxisomal acyl-coenzyme A oxidase 1, whose first pA site was in the ORF, the second in the 3′UTR (Figure 5B). If it mainly uses the first tailing site, the protein sequence will be shortened, which may change the function of the protein. LOC118264954 was found to be cadherin-related tumor suppressor-like, whose first pA site is in an intron, and another one is in 3′UTR (Figure 5C). If it mainly uses the first tailing site, the protein sequence will be shortened, which may change the function of the protein. LOC118275420 was Acyl-CoA dehydrogenase (Figure 5D). Both pA sites were in 3′UTR. Its variable tailing does not lead to different protein sequences, but it contains many regulatory elements, such as miRNA recognition sites, in order to alter the length of 3′UTR.

## 4. Discussion

With the rapid development of high-throughput sequencing technology, researchers have obtained massive amounts of sequencing data, including genomic and transcriptome, which allows researchers to explore the regulation mechanism of genes. In this study, a complete collection of the poly(A) site was performed, followed by a preliminary study of its polyadenylation signal, polyadenylation, and gene regulation properties at the APA level. This study lays the foundation for further research on the RNA modification of polyadenylation and the genetic regulation of APA in *Spodoptera frugiperda*.

As described previously [35], the cleavage and polyadenylation sites of *Spodoptera frugiperda* were identified to perform the first analysis of PAS usage. To achieve it, over 10 million individual tags (28,931) and reads (10,728,582) were mapped, and all of the 50,516 CSs were identified. Although the genome of *Spodoptera frugiperda* has been annotated, the various isoforms produced by APA could not be distinguished by it. A major finding is that APA is widely used in *Spodoptera frugiperda*.

There are currently many methods for identifying cleavage and polyadenylation sites [6,36,37,38], but these require specific sequencing protocols. In previous studies, a large amount of transcriptome and EST data has been produced. Therefore, we could conduct further analysis with these data and obtain more useful information about polyadenylation. Tian’s study, for example, found 29,823 poly(A) sites in humans and 16,282 poly(A) sites in mouse [11]. We identified 25,699 poly(A) sites in *Spodoptera frugiperda*, which is a reasonable number based on the number of genes in the genome. This study found that in *Spodoptera frugiperda*, many mRNAs have different gene isoforms, because the location of the cleavage is different. The results showed that APA regulation has a significant impact on regulating gene expression of *Spodoptera frugiperda*.

APA has an effect on the fate of mRNA, and longer UTRs are unfavorable for gene expression because APA can provide more sites for miRNA-binding [39] RNA-binding recognition proteins. The result shows that the distribution of PAS variants was consistent with that of human and fly [12]. The PAS usage frequency of AAUAAA/AUUAAA is similar to that of other PAS variants, and all of these sites are used for CPSF recognition. This indicates that in different animals the polyadenylation process is relatively conserved. Interestingly, the previously reported PAS sequences upstream of the cleavage sites were not found in 10.13% of transcripts of *Spodoptera frugiperda* [10]. The lost signals indicate there are alternative PAS sequences or an alternative mechanism that has not been identified. Compared with Beaudiong and Tian’s study [10,11], this result shows the higher frequency of unidentified PAS and the lower ratio of known PAS variants. However, such a result can be affected by more than one factor because the basis of this analysis is RNA-seq data, whose quality is essential for PAS analysis. What is more, this study utilized more data than the previous studies. In addition, the available scale of PAS sequencing data of human and mouse is much richer.

Through GO analysis and KEGG analysis of the APA gene of *Spodoptera frugiperda*, it can be seen that the APA gene-coding protein is widely distributed in cells and has many mediated pathways. The results of GO analysis showed that the encoded proteins were mostly used to perform two biological functions: binding and catalysis. KEGG results showed that the APA gene was enriched in various pathways related to cell metabolism. Through research, it can be found that the APA gene has a wide range of functions. The APA mechanism leads to the same gene producing different mRNA under different conditions, which is conducive to the normal progress of life activities and adaptation to environmental changes.

The research has significant future directions. Although we identified 50,616 poly(A) sites, only 59.22% of them could be mapped to genes. The insufficient and unclear annotation of the *Spodoptera frugiperda* genome data may be the reason for this phenomenon. Another reason may be that the full-length transcriptome data only comes from the brain tissue of the larva, and there is no tissue source of other instars and other tissues, which leads to some transcripts not being expressed in the brain tissue, and thus the polyadenylation site cannot be found. In a specific tissue, the usage of polyadenylation sites may show certain rules. For example, short transcripts with early pAs are common in testis, as shown in *Drosophila melanogaster* [21], which is also the reason why the poly(A)sites we identified are not comprehensive. Moreover, the usage of tailing sites in brain tissue should be explored in future studies. Therefore, more transcriptome data information of different *Spodoptera frugiperda* tissues is needed to solve these problems.

## 5. Conclusions

In this study, we first identified 50,616 pA sites on the *Spodoptera frugiperda* and analyzed them. We also analyzed the distribution and frequency of nucleotides and motifs within 100 bp upstream and downstream of 50,616 pA sites to identify the core cis-acting elements that define these pA sites. The results showed that 51.64% of the 50,616 pA sites had the conserved AAUAAA hexamers, while 10.13% of pA sites had none of the AAUAAA-like hexamers. Among these genes, more than 64.76% have more than one pA site. The PAS data provided by this paper will help to improve the annotation of *Spodoptera frugiperda* genome and facilitate the research of *Spodoptera frugiperda*. In addition, the results of this experiment can be used as a basis for the comparative analysis of polyadenylation signal between different species.

## Figures and Tables

**Table 1:** Summary of all mRNA transcripts extracted from the EST and full-length transcriptome databases of *Spodoptera frugiperda*.

Types	EST	Full-Length Transcriptome
Raw reads	65,423	7,510,007,630
Poly(A) tail-containing reads	35,853	375,500,381
Reads mapped to the genome	28,931	10,728,582
pA sites	10,245	40,371
Total pA cluster	50,616	

**Table 2:** Frequency (%) of hexamers motifs.

Hexamers	Frequency (%)
AAUAAA	51.64
AUUAAA	12.15
UAUAAA	4.38
AGUAAA	4.17
AAGAAA	2.63
AAUAUA	2.42
AAUACA	2.32
CAUAAA	2.00
GAUAAA	2.01
AAUGAA	1.64
UUUAAA	1.25
ACUAAA	1.19
AAUAGA	1.05
AAAAAG	1.03
Other	10.13

## Data Availability

Full-length transcriptome data of *Spodoptera frugiperda* were downloaded from NCBI, and the accession numbers are SRR14569375.1, SRR14569378.1, SRR14621792.1. EST data of *Spodoptera frugiperda* were on Supplementary Materials Dataset S1.

## Supplementary Materials

The following supporting information can be downloaded at: https://www.mdpi.com/article/10.3390/insects13090803/s1. Dataset S1: All 65,423 Spodoptera frugiperda EST transcripts. Table S1: All 50,616 pA sites genome position. Script S1: The perl script used in extract the polyA transcript. Figure S1: Sequence characterization around the cleavage site. Figure S2: The genes with 1 polyadenylation signals were applied for GO annotation and enrichment. Figure S3: The genes with 3 or more polyadenylation signals were applied for GO annotation and enrichment.

## References

[1] Chen Z., Li Y., Krug R.M. (1999). Influenza A virus NS1 protein targets poly(A)-binding protein II of the cellular 3′-end processing machinery. EMBO J..

[2] Zhang Y., Song J., Zhang M., Deng Z. (2022). Analysis Polyadenylation Signal Usage in *Sus scrofa*. Animals.

[3] Jolles B., Jean-Jean O. (2021). Poly(a) tail degradation in human cells: ATF4 mrna as a model for biphasic deadenylation. Biochimie.

[4] Xu C., Zhang J. (2018). Alternative Polyadenylation of Mammalian Transcripts Is Generally Deleterious, Not Adaptive. Cell Syst..

[5] Libri D. (2015). Endless Quarrels at the End of Genes. Mol. Cell.

[6] Elkon R., Ugalde A.P., Agami R. (2013). Alternative cleavage and polyadenylation: Extent, regulation and function. Nat. Rev. Genet..

[7] Chen W., Jia Q., Song Y., Fu H., Wei G., Ni T. (2017). Alternative polyadenylation:methods, findings, and impacts. J. Genom. Proteom. Bioinform..

[8] Fitzgerald M., Shenk T. (1981). The sequence 5′-AAUAAA-3′forms parts of the recognition site for polyadenylation of late SV40 mRNAs. Cell.

[9] Burri D., Zavolan M. (2021). Shortening of 3′ UTRs in most cell types composing tumor tissues implicates alternative polyadenylation in protein metabolism. RNA.

[10] Beaudoing E., Freier S., Wyatt J.R., Claverie J.M., Gautheret D. (2000). Patterns of variant polyadenylation signal usage in human genes. Genome Res..

[11] Tian B., Hu J., Zhang H., Lutz C.S. (2005). A large-scale analysis of mRNA polyadenylation of human and mouse genes. Nucleic Acids Res..

[12] Retelska D., Iseli C., Bucher P., Jongeneel C.V., Naef F. (2006). Similarities and differences of polyadenylation signals in human and fly. BMC Genom..

[13] Lakshmanan V., Bansal D., Kulkarni J., Poduval D., Krishna S., Sasidharan V., Anand P., Seshasayee A., Palakodeti D. (2016). Genome-Wide Analysis of Polyadenylation Events in Schmidtea mediterranea. G3.

[14] Hoque M., Ji Z., Zheng D., Luo W., Li W., You B. (2013). Analysis of alternative cleavage and polyadenylation by 3′ region extraction and deep sequencing. Nat. Methods.

[15] Zhang Y., Sun Y., Shi Y., Walz T., Tong L. (2019). Structural Insights into the Human Pre-mRNA 3′-End Processing Machinery. Mol. Cell.

[16] Shen Y., Ji G., Haas B.J., Wu X., Zheng J., Reese G.J., Li Q.Q. (2008). Genome level analysis of rice mRNA 3′-end processing signals and alternative polyadenylation. Nucleic Acids Res..

[17] Li Y., Schaefke B., Zou X., Zhang M., Heyd F., Sun W., Zhang B., Li G., Liang W., He Y. (2020). Pan-tissue analysis of allelic alternative polyadenylation suggests widespread functional regulation. Mol. Syst. Biol..

[18] Guvenek A., Tian B. (2018). Analysis of alternative cleavage and polyadenylation in mature and differentiating neurons using RNA-seq data. Quant. Biol..

[19] Tian B., Manley J.L. (2013). Alternative cleavage and polyadenylation: The long and short of it. Trends Biochem. Sci..

[20] Neve J., Furger A. (2014). Alternative polyadenylation: Less than meets the eye?. Biochem. Soc. Trans..

[21] Smibert P., Miura P., Westholm J.O., Shenker S., May G., Duff M.O., Zhang D., Eads B.D., Carlson J., Brown J.B. (2012). Global patterns of tissue-specific alternative polyadenylation in Drosophila. Cell Rep..

[22] Chartier A., Joly W., Simonelig M. (2017). Measurement of mRNA Poly(A) Tail Lengths in Drosophila Female Germ Cells and Germ-Line Stem Cells. Methods Mol. Biol..

[23] Misof B., Liu S., Meusemann K., Peters R.S., Donath A., Mayer C., Frandsen P.B., Ware J., Flouri T., Beutel R.G. (2014). Phylogenomics resolves the timing and pattern of insect evolution. Science.

[24] Montezano D.G., Specht A., Sosa-Gomez D.R. (2018). Host plants of *Spodoptera frugiperda* (Lepidoptera: Noctuidae) in the Americas. Afr. Entomol..

[25] FAO (2018). Fall armyworm likely to spread from India to other parts of Asia with South East Asia and South China most at risk. Rome: Food and Agriculture Organization of United Nations.

[26] Yang X., Liu Y., Luo M., Li J., Wang W., Wan J., Jiang J. (2019). The first discovery of *Spodoptera frugiperda* in Jiangcheng County, Yunnan Province. Yunnan Agric..

[27] Kakumani P.K., Malhotra P., Mukherjee S.K. (2014). A draft genome assembly of the army worm, *Spodoptera frugiperda*. Genomics.

[28] Gouin A., Bretaudeau A., Nam K. (2017). Two genomes of highly polyphagous lepidopteran pests (*Spodoptera frugiperda*, Noctuidae) with different host-plant ranges. Sci. Rep..

[29] Nandakumar S., Ma H., Khan A.S. (2017). Whole-genome sequence of the *Spodoptera frugiperda* Sf9 insect cell line. Microbiol. Resour. Announc..

[30] Liu H., Lan T., Fang D., Gui F., Wang H., Guo W., Chen X., Chang Y., He S., Lyu L. (2019). Chromosome level draft genomes of the fall armyworm, *Spodoptera frugiperda* (Lepidoptera: Noctuidae), an alien invasive pest in China. BioRxiv.

[31] Xin Y., Yi Y., Yang M., Hua X., Fei L.I. (2019). The genome annotation and comparative genomics analysis of *spodoptera frugiperda*. J. Environ. Entomol..

[32] Xiao H., Ye X., Xu H., Mei Y., Yang Y., Chen X., Yang Y., Liu T., Yu Y., Yang W. (2020). The genetic adaptations of fall armyworm Spodoptera frugiperda facilitated its rapid global dispersal and invasion. Mol. Ecol. Resour..

[33] Zhang L., Liu B., Zheng W., Liu C., Zhang D., Zhao S., Li Z., Xu P., Wilson K., Withers A. (2020). Genetic structure and insecticide resistance characteristics of fall armyworm populations invading China. Mol. Ecol. Resour..

[34] Gimenez S., Abdelgaffar H., Goff G.L., Hilliou F., Blanco C.A., Hänniger S., Bretaudeau A., Fuentes J.L., Legeai F. (2020). Adaptation by copy number variation increases insecticide resistance in the fall armyworm. Commun. Biol..

[35] Yang L., Xing B., Li F., Wang L.K., Yuan L., Mbuji A.L., Peng Z., Malhat F., Wu S. (2021). Full-length transcriptome analysis of *Spodoptera frugiperda* larval brain reveals detoxification genes. PeerJ.

[36] Pickrell J.K., Marioni J.C., Pai A.A., Degner J.F., Engelhardt B.E., Nkadori E., Veyrieras J.B., Stephens M., Gilad Y., Pritchard J.K. (2010). Understanding mechanisms underlying human gene expression variation with RNA sequencing. Nature.

[37] Arefeen A., Xiao X., Jiang T. (2019). DeepPASTA: Deep neural network based polyadenylation site analysis. Bioinformatics.

[38] Jafar Z., Tariq S., Sadiq I., Nawaz T., Akhtar M.N. (2019). Genome-Wide Profiling of Polyadenylation Events in Maize Using High-Throughput Transcriptomic Sequences. G3.

[39] Fabian M.R., Sonenberg N., Filipowicz W. (2010). Regulation of mRNA translation and stability by microRNAs. Annu. Rev. Biochem..

