# Supplementary material for: Analysis of Polyadenylation Signal Usage with Full-Length Transcriptome in Spodoptera frugiperda (Lepidoptera: Noctuidae)
Source: Insects. 2022 Sep 2;13(9):803. doi: 10.3390/insects13090803 (PMC9505298; doi:10.3390/insects13090803)

Figure S1. Sequence characterization around the cleavage site. (A) Distribution frequency of the dinucleotide within 100 bp upstream and downstream of the cleavage site. (B) Number of hexamers within the 100 bp of the cleavage site. The number of genes is shown on the Y-axis. The position zero is the CS.

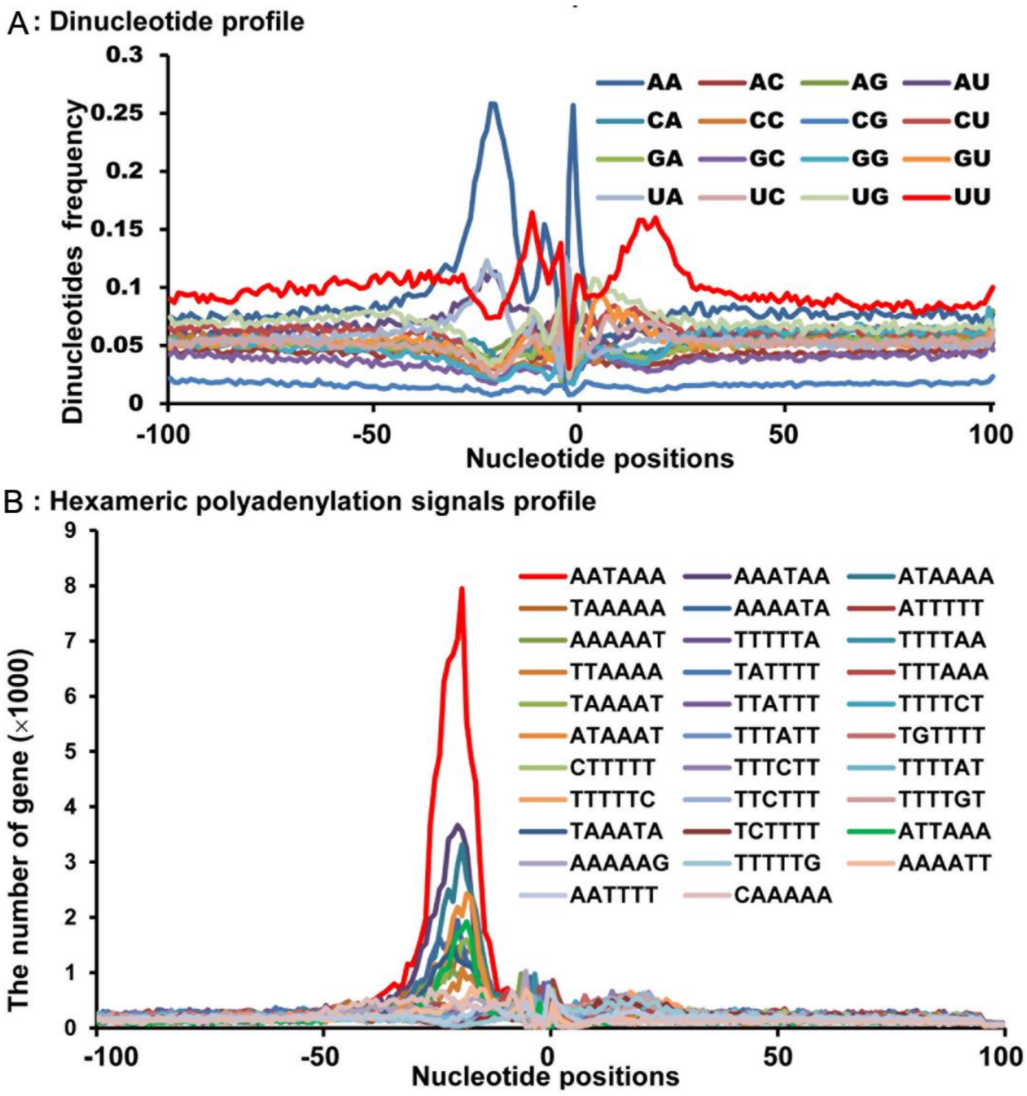

Figure S2. The genes with 1 polyadenylation signals were applied for GO annotation and enrichment.

A

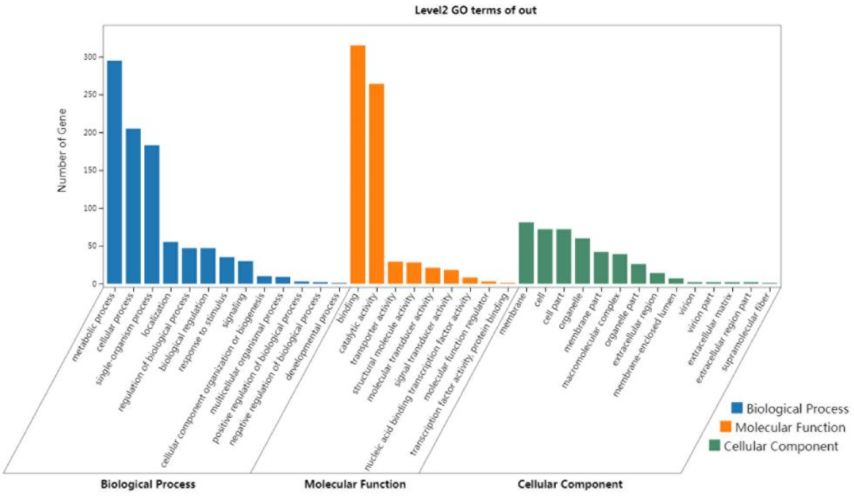

B

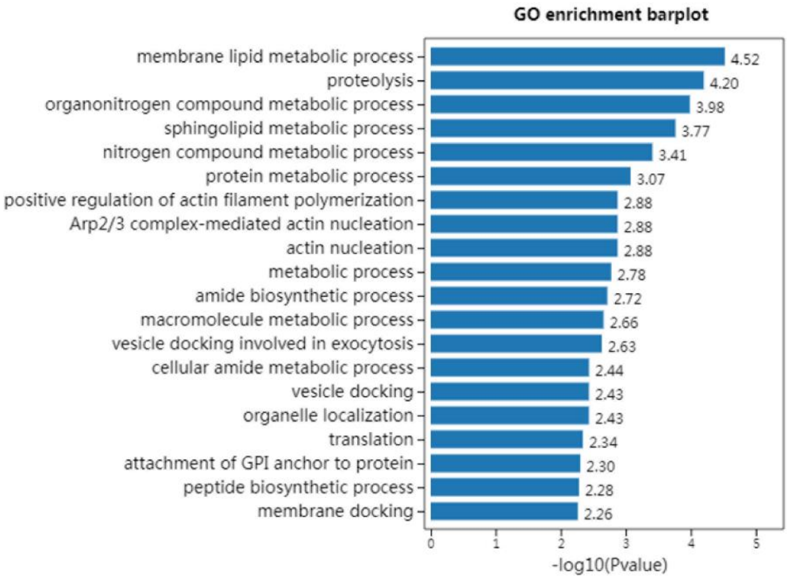

Figure S3. The genes with 3 or more polyadenylation signals were applied for GO annotation and enrichment.

A

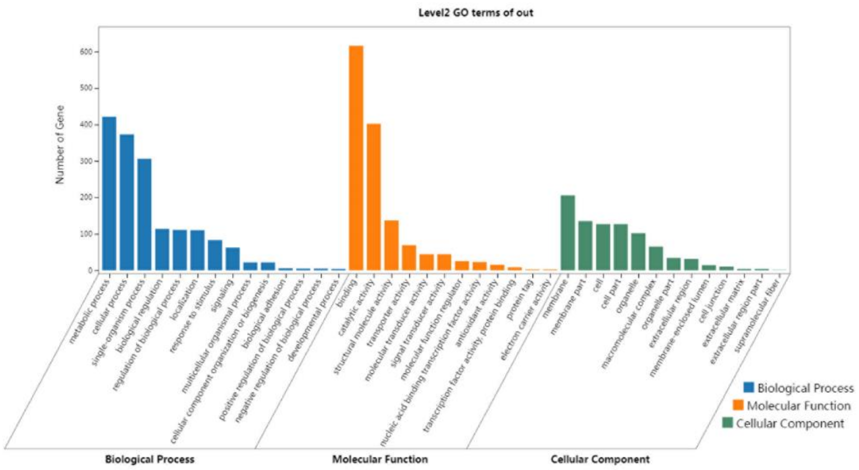

B

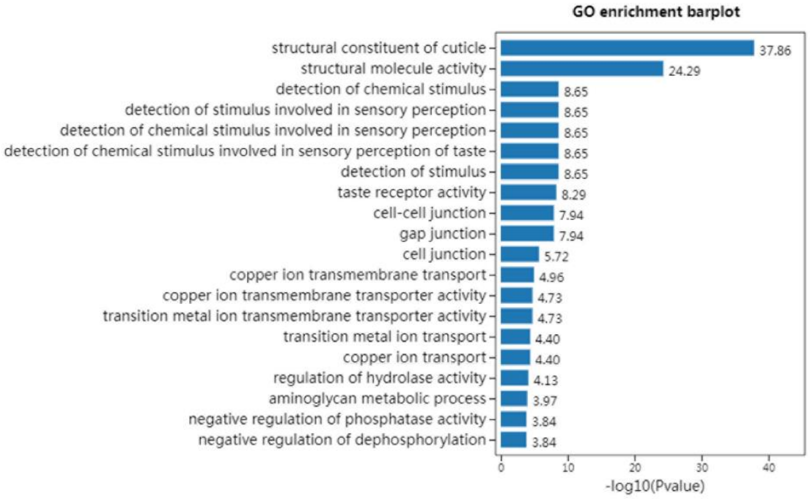

Supplement: Supplementary file 1 [file insects-13-00803-s001.zip › Figure S1-S3.pdf]
